# ITTHACA: A Joint Multicenter Initiative in the Basque Country (Spain) for Biomarker, Biosensor, and Predictive Model Research for Healthy Aging based on CITA GO‐ON study, a FINGER Study

**DOI:** 10.1002/alz70860_099250

**Published:** 2025-12-23

**Authors:** Iñigo Tellaetxe Elorriaga, Eukene Imatz, Bergoi Ibarlucea, Ainara Cano, Esther Sanmartín, Itziar Tueros, Unai Ayala, Arantxa Gonzalez de Heredia, Carla Zaldua, Jose Luis Zugaza, Ignacio Torres Aleman, Maite Garcia‐Sebastian, Pablo Martínez‐Lage, Asier Erramuzpe

**Affiliations:** ^1^ Biobizkaia HRI, Barakaldo, Bizkaia, Spain; ^2^ University of the Basque Country, Leioa, Bizkaia, Spain; ^3^ Tecnalia, Basque Research and Technology Alliance (BRTA), Bilbao, Bizkaia, Spain; ^4^ AZTI, Food Research, Basque Research and Technology Alliance (BRTA), Bilbao, Bizkaia, Spain; ^5^ Mondragon Goi Eskola Politeknikoa, Mondragon Unibertsitatea, Arrasate, Gipuzkoa, Spain; ^6^ accexible, Barcelona, Spain; ^7^ Achucarro Basque Center for Neuroscience, Leioa, Spain; ^8^ Ikerbasque, the Basque Foundation for Science, Bilbao, Bizkaia, Spain; ^9^ CITA Alzheimer Foundation, Donostia ‐ San Sebastián, Gipuzkoa, Spain; ^10^ Fundación CITA‐Alzhéimer Fundazioa, Donostia‐San Sebastian, Spain

## Abstract

**Background:**

The ITTHACA project is a collaborative initiative involving six research institutions from the Basque Country including Universities, Health, Technology and Basic Research Institutions. It builds upon the ongoing CITA GO‐ON) CITA Go‐On study, ClinicalTrials.gov, NCT04840030) cohort study, which adapts the Finnish FINGER [Ngandu, T., et al. 2015] model to the local context. ITTHACA focuses on enhancing healthy aging by identifying markers, prediction models and sensors for in vivo monitoring that allow the establishment and implementation of combined intervention strategies in the population.

**Method:**

This one‐year randomized‐controlled trial (total *n* = 250; 125 control and 125 intervention), focused on 60‐85‐year‐old males and females at risk of dementia, adopts a multimodal approach. Biomarker identification includes proteomics and metabolomics in biological fluids (blood) and 16S metagenomics and lipidomics in the gut microbiome (stool), as well as employing a FINGER‐like mice model. Biosensor technology under development includes multi‐channel bioimpedance spectroscopy for tissue analysis and electrochemical sensors for real‐time detection of aging markers in biofluids. Predictive modeling integrates data from these analyses and multiple domains—cognition, cardiovascular health, voice, food texture perception and habits—to generate diagnostic tools that monitor biological aging and inform early interventions. A proof‐of‐concept study in an older population sample, with special attention to user experience, will evaluate the potential benefits of these findings in improving the quality of life for older adults.

**Result:**

Not applicable. The ITTHACA project is ongoing, with outcomes expected to include validated biomarkers, novel biosensors, and predictive models that facilitate early interventions.

**Conclusion:**

ITTHACA demonstrates the power of interdisciplinary collaboration in tackling the complex multidomain challenge of aging. By leveraging the expertise of complementary Basque Country Research Centers, this initiative is poised to produce innovative resources for prolonging healthy and autonomous living. The project's outcomes are expected to support new therapeutic strategies and socio‐healthcare interventions that address the rising prevalence of aging‐related conditions, including cognitive decline.

